# Is the hydrophobic core a universal structural element in proteins?

**DOI:** 10.1007/s00894-017-3367-z

**Published:** 2017-06-16

**Authors:** Barbara Kalinowska, Mateusz Banach, Zdzisław Wiśniowski, Leszek Konieczny, Irena Roterman

**Affiliations:** 1 Department of Bioinformatics and Telemedicine, Jagiellonian University – Medical College, Lazarza 16, 31-530 Krakow, Poland; 2 Faculty of Physics, Astronomy and Applied Computer Science, Jagiellonian University, Łojasiewicza 11, 30-348 Krakow, Poland; 3 Chair of Medical Biochemistry, Jagiellonian University – Medical College, Kopernika 7, 31-034 Krakow, Poland

**Keywords:** Protein folding, Hydrophobicity, Hydrophobic core

## Abstract

The hydrophobic core, when subjected to analysis based on the fuzzy oil drop model, appears to be a universal structural component of proteins irrespective of their secondary, supersecondary, and tertiary conformations. A study has been performed on a set of nonhomologous proteins representing a variety of CATH categories. The presence of a well-ordered hydrophobic core has been confirmed in each case, regardless of the protein’s biological function, chain length or source organism. In light of fuzzy oil drop (FOD) analysis, various supersecondary forms seem to share a common structural factor in the form of a hydrophobic core, emerging either as part of the whole protein or a specific domain. The variable status of individual folds with respect to the FOD model reflects their propensity for conformational changes, frequently associated with biological function. Such flexibility is expressed as variable stability of the hydrophobic core, along with specific encoding of potential conformational changes which depend on the properties of helices and β-folds.

## Introduction

The traditional classification of secondary and supersecondary protein structures covers helical and β-fragments along with their mutual arrangement within the protein body. This α/β classification varies with respect to the participation of both folds in proteins, as well as their orientation. Purely helical (hemoglobin, cytochrome) or purely β-shaped (immunoglobulins) structures, as well as proteins characterized by near-equal participation of both types (lactate dehydrogenase or carboxypeptidase), can be identified in protein databases (e.g., PDB). The supersecondary structure, which determines the mutual arrangement of secondary folds can be expressed as follows (the following list is derived from a commonly used biochemistry textbook [1]):

1. βαβ – helical fragment linking two parallel β-strands
2. β hairpin – two anti-parallel β-strands linked by a tight U-turn loop
3. αα motif – two successive helices linked by a tight U-turn loop
4. Greek key motif – β-strands following one another in an arrangement reminiscent of classic Greek ornaments
5. β barrels – coaxial β-strands forming a cyclical system reminiscent to the arrangement of planks forming a wooden barrel.

Attempts to introduce a detailed structural classification of domain units in large proteins have resulted in the creation of the CATH/Gene3D (http://www.cathdb.info/ 27 Apr 2016) database, which currently (as of 27 April 2016) contains 26 million domains arranged into 2738 families. Structural classification is performed automatically upon inclusion of a new protein in PDB, via an exhaustive search for proteins homologous to the new entrant [2]. It is immediately apparent that the number of distinct families is quite high, notwithstanding the overall size of the database. This suggests that protein structures are diverse and difficult to classify.

The concept of biological activity likewise remains a mystery [3], despite successful attempts to program it into synthetic proteins. An in-depth analysis of the stability of the backbone and side chains facilitating stabilization of one of two possible rotametric forms, is presented in [4]. The great diversity of geometric forms makes it difficult to propose a single, common classification based on a unified criterion. This, however, does not imply that attempts to identify such a criterion are futile.

Our work focuses on proteins characterized by major supersecondary variability and proposes a coherent classification, covering all cases. The starting point is to acknowledge the common environment in which all proteins undergo folding and gain biological function — water. Water is the immanent condition ensuring proper folding and biological activity. This is why the water participation and its influencing on protein structure is the basis for the fuzzy oil drop model applied to interpret the different structures of proteins. We assume that the proposed model visualizes the effects of the influence of water environment despite the lack of knowledge of its own structuralization.

Our analysis of diverse structures is based on the fuzzy oil drop model and shows that both secondary and supersecondary structural motifs participate in the formation of a domain- or protein-wide hydrophobic core. In addition to enzymes (which are the most widely represented group), the work also discusses proteins capable of binding specific ligands (cytochrome) as well as antigen-binding proteins (immunoglobulin).

We establish that each of the presented molecules (or domains) contains a “seed” (of variable size) representing its hydrophobic core, which, according to textbook knowledge, is responsible for tertiary structural stabilization. As shown in our other papers, local deviations from the “idealized” hydrophobic core structure (i.e., high hydrophobicity density in the central part of the protein body and low hydrophobicity density on the surface) are often linked to the protein’s biological activity. The presence of cavities or exposure of hydrophobic residues on the surface creates suitable conditions for ligand binding and protein complexation respectively. Eliminating such discrepancies from analysis of the hydrophobic core structure invariably leads to identification of parts of the molecule for which the idealized distribution of hydrophobicity closely corresponds to observed values. Such parts are thought to mediate structural stabilization of the protein (or domain) as a whole [5–9].

In this work we show that, regardless of secondary and supersecondary conformational properties, the presence of a more or less prominent hydrophobic core is a common phenomenon. We identify proteins where the core is highly consistent with theoretical values (3D Gaussian) as well as those which exhibit local deviations from the theoretical model, typically associated with the capability to bind ligands or attract complexation partners. The study set is very diverse, yet exhibits common characteristics which point to the necessary presence of water in their environment — this applies to many different molecules encountered in the cell, but particularly to proteins.

Restricting protein structure analysis to topological aspects would disregard the most important factor — the influence of the water environment, which plays an active and often decisive role in the folding process. Hence, our study of the hydrophobic core — a product of the environment, which determines the biological activity of proteins.

Water environment is treated as the external force field which together with the internal force field (non-bonding interaction) participates in structuralization process and ensures the biological activity. The presented paper is an attempt to show the possible interpretation of structural effects which may express the role of water.

## Materials and methods

### Protein database

Our analysis is based on a set of proteins derived from a popular biochemistry textbook [1]. Table 1 reveals the structural variability observed at the supersecondary level.

**Table 1:** Summary of proteins subjected to analysis

Protein	Name	Source	Class	Ref.
	α/β-domain fold twisted β-sheet			
1A5Z	Lactate dehydrogenase	Bacteria	αβ complex	(Auerbach et al. [10])
1FW8	Phosphoglycerate kinase	Yeast	α/β 3-layer (aba)	(Tougard et al. [11])
	α,β-domain fold			
1AMK	Triose phosphate isomerase	Bacteria	α β barrel	(Williams et al. [12])
4DRS	Pyruvate kinase	Bacteria	α/β 3-layer (aba) α/β barrel mainly β barrel	(Cook et al. [13])
	All β-domain super-fold			
1B4L	Superoxide dismutase	Yeast	Mainly β sandwich	(Hart et al. [14])
1CON	Concanavalin	Plant	Mainly β sandwich	(Naismith et al. [15])
	β-barrel			
1RBP	Retinol binding	Human	Mainly β barrel	(Cowan et al. [16])
1PNG	Aspargine amidase	Bacteria	Mainly β sandwich	(Kuhn et al. [17])
1TIM	Triose phosphatase isomerase	Chicken	α β barrel	(Banner et al. [18])
	Miscellaneous			
256B	Cytochrome	E-coli	α Up-down bundle	(Lederer et al. [19])
7FAB	Immunoglobulin	Human	Mainly β sandwich	(Saul and Poljak [20])
6LDH	Lactate dehydrogenase	Bacteria	α β complex	(Abad-Zapatero et al. [21])
	Cytochrome fold			
155C	Cytochrome	Bacteria	α orthogonal bundle	(Timkovich and Dickerson [22])
1JDL	Cytochrome	Bacteria	α orthogonal bundle	(Camara-Artigas et al. [23])
2C2C	Cytochrome	Bacteria	α orthogonal bundle	(Bhatia –PDB [24])
5CYT	Cytochrome	Fish	α orthogonal bundle	(Takano [25])
4 J20	Cytochrome	Bacteria	α orthogonal bundle	(Yu et al. [26])

The selection is based on supersecondary structural order. The study set is consistent with the one proposed in (Devlin 2011).

### The fuzzy oil drop model

An in-depth presentation of the fuzzy oil drop model can be found in an Open Access publication [27, 28]. In the interest of saving space, we will limit ourselves to recapitulating its basic tenets.

The fuzzy oil drop model is a modification of the oil drop model originally introduced by Kauzmann [29]. The original model was discrete, distinguishing a central hydrophobic part and an outer hydrophilic shell. In contrast, the fuzzy oil drop model introduces a 3D Gaussian which peaks at the center of the encapsulating ellipsoid, with values decreasing along with distance from the center, reaching near 0 at a distance of 3σ (where σ is a parameter of the Gaussian expressing the size of the ellipsoid). Distribution along each axis can be characterized by an appropriate sigma parameter. The values of such a function are assumed to represent theoretical (idealized) distribution of hydrophobicity in a protein molecule.

The actual (observed) hydrophobicity distribution depends on the location of each residue in the protein body (in our calculations we apply the function proposed by Levitt [30]). Each residue (represented by its effective atom — averaged-out positions of all atoms belonging to a particular residue) aggregates hydrophobic interactions with other residues separated from it by not more than 9 Å. Clearly, the observed distribution may differ from the idealized (Gaussian) profile. The scope of such interactions is dependent on the relative proximity between both residues and on their intrinsic hydrophobicity, which can be measured experimentally or predicted on theoretical grounds. Different hydrophobicity scales can be applied (a comparison of results obtained for two different scales is presented in [31]). Normalization of both distributions (theoretical and observed) facilitates meaningful comparisons: in particular, we can identify residues for which theoretical (T) values diverge significantly from their observed (O) counterparts.

Both hydrophobicity distribution profiles — expected (T) and observed (O) — can be compared quantitatively. Quantitative expression of the differences between the expected (T) and observed (O) distribution is possible by using the Kullback-Leibler divergence entropy formula [32]:

$$ {D}_{KL}\left( p\left|{p}^0\right.\right)=\sum_{i=1}^N{p}_i{ \log}_2\left({p}_i/{p}_i^0\right) $$

The value of *D*_*KL*_ expresses the distance between the observed (*p*) and target (*p*_*0*_) distributions, the latter of which is given by the 3D Gaussian (T). The observed distribution (*p*) is referred to as O.

For the sake of simplicity, we introduce the following notation:

$$ O\left| T\right.=\sum_{i=1}^N{O}_i{ \log}_2\left({O}_i/{T}_i\right) $$

Since *D*_*KL*_ is a measure of entropy it must be compared to a reference value. In order to facilitate meaningful comparisons, we have introduced another opposite boundary distribution (referred to as “uniform” or R) which corresponds to a situation where each effective atom possesses the same hydrophobicity density (1/*N*, where *N* is the number of residues in the chain). This distribution is deprived of any form of hydrophobicity concentration at any point in the protein body:

$$ O\left| R\right.=\sum_{i=1}^N{O}_i{ \log}_2\left({O}_i/{R}_i\right) $$

Comparing O|T and O|R tells us whether the given protein (O) more closely approximates the theoretical (T) or uniform (R) distribution. Proteins for which O|T > O|R are regarded as lacking a prominent hydrophobic core. To further simplify matters we introduce the following relative distance (RD) criterion:

$$ RD= O\left| T\right|\left( O\left| T+ O\right| R\right) $$

**Fig. 1:**
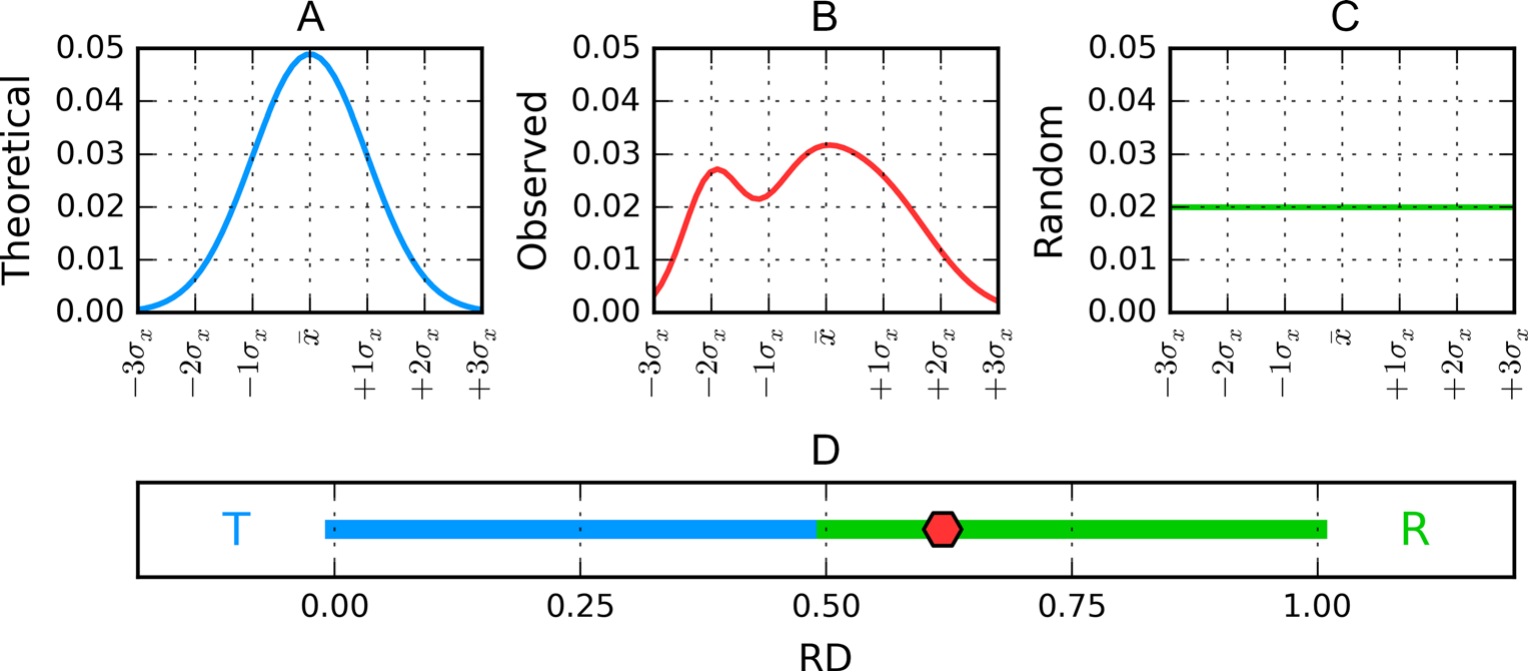
Graphical representation of fuzzy oil drop model hydrophobicity distributions obtained for a hypothetical protein reduced to a single dimension for simplicity. A is the theorized Gaussian distribution (blue) while chart C corresponds to the uniform distribution (green). Actually observed (red) hydrophobicity density distribution in the target protein B, while its corresponding value of RD (relative distance) in D is marked on the horizontal axis with a red diamond. According to the fuzzy oil drop model this protein does not contain a well-defined hydrophobic core, because its RD value, equal to 0.619, is above the 0.5 threshold (or, generally, closer to R than to T)

RD < 0.5 is understood to indicate the presence of a hydrophobic core. Figure 1 presents a graphical representation of RD values, restricted (for simplicity) to a single dimension.

*D*_*KL*_ (as well as O|T, O|R and RD) may be calculated for specific structural units (protein complex, single molecule, single chain, selected domain etc.) In such cases the bounding ellipsoid is restricted to the selected fragment of the protein. It is also possible to determine the status of polypeptide chain fragments within the context of a given ellipsoid. This procedure requires prior normalization of O and R distributions describing the analyzed fragment, whose length is denoted as N. Note that any selected fragment must be of a reasonable length — calculations cannot be performed for an individual residue.

Generally the idealized distribution (T) is treated as the target for O|T calculation, while the unified distribution (R) is used when computing O|R.

The above procedure will be applied in the analysis of proteins described in this paper. By restricting our analysis to individual fragments, we can determine whether a given fragment participates in the formation of a hydrophobic core. In particular, fragments representing well-defined secondary folds which satisfy RD < 0.5 are thought to contribute to structural stabilization, while fragments for which RD > = 0.5 are less stable. Such fragments, if present on the surface of the protein, may potentially form complexation sites. Specific fragments are derived by analyzing the protein’s secondary conformation. Identification of secondary folds and the composition of protein domains follows the CATH [33] and PDBsum [34] classifications. Likewise, inter-domain/inter-chain contacts have been identified on the basis of the PDBsum distance criteria [34].

A graphical presentation of RD interpretation is shown in Fig. 1.

## Results

### α/β-domain fold twisted β-sheet

This category is represented by the nonhomologous domain 1 of lactate dehydrogenase (1A5Z – domain 1) and phosphoglycerate kinase (1FW8 – domain 2). The α,β-superfold can be described as a central twisted β-sheet surrounded by a ring of helices. RD values for both domains and their individual secondary folds are listed in Table 2.

**Table 2:** Structural properties of the hydrophobic core in two proteins representing the α/β-domain fold twisted β-sheet category

Lactate dehydrogenase (1A5Z-D1) 3.40.50.720 α-β 3-layer (aba) sandwich			Phosphoglycerate kinase (1FW8-D2) 3.40.50.1260 α-β 3-layer (aba) sandwich		
Structure	Fragment	RD	Structure	Fragment	RD
Domain 1		0.416	**Domain 2**		**0.523**
β-sheet	Parallel	0.298	β**-sheet I and II**		**0.516**
Helices		**0.530**	**Helices**		**0.502**
Helices No 68–84	No ligand binding res.	0.449	β-sheet I	Parallel	0.492
β-conform.	22–28	0.198	β-conform.	133–140	0.393
	47–52	0.259		158–163	0.127
	77–83	0.331		205–207	0.145
	92–97	0.180		**210–216**	**0.706**
	134–138	0.243		223–229	0.455
	159–163	0.307		**238–242**	**0.763**
				258–263	0.426
Helices	30–44	0.498		294–298	0.144
	**55–70**	**0.589**			
	**71–73**	**0.980**	Helices	146–155	0.353
	84–89	0.366		166–175	0.244
	108–131	0.444		185–202	0.347
	141–154	0.365		243–257	0.358
				271–275	0.363
				**276–291**	**0.721**
				300–309	0.378
				311–315	0.228
			Domain 2	No β-II	0.492
			β-sheet I No 294–298	No ligand binding	0.320
			**β-sheet II**	**Anti-parallel**	**0.625**
			Helices No 287–301	No ligand binding	0.455

Values listed in boldface correspond to RD > 0.5. Items labeled “No (…)” represent shortened fragments where elimination of the indicated residues changes the relation to RD < 0.5. “Ligand binding” stands for fragments directly involved in ligand binding. Our experience with the fuzzy oil drop model indicates that such fragments often exhibit significant deviations from the theoretical hydrophobicity distribution.

**Fig. 2:**
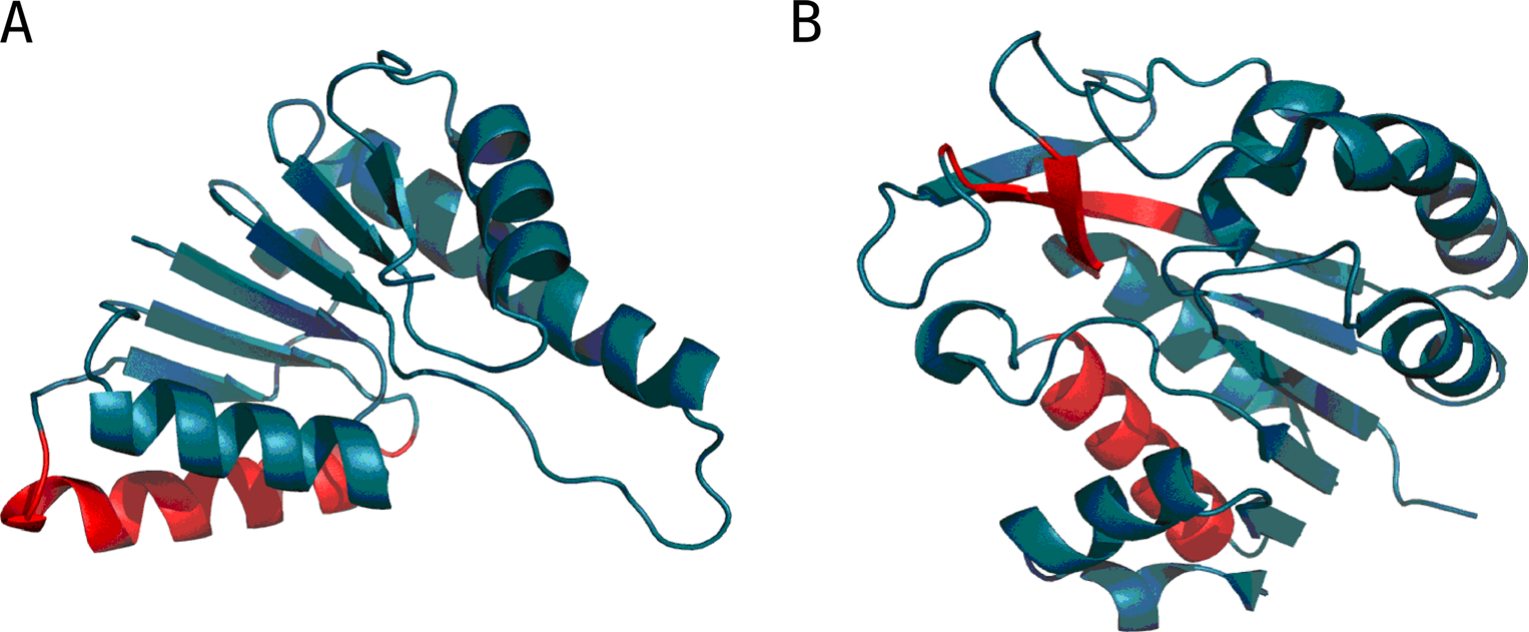
3D representation of 1A5Z-D1 (A) and 1FW8-D2 (B). Fragments marked in red diverge from the theoretical model (RD > 0.5)

**Fig. 3:**
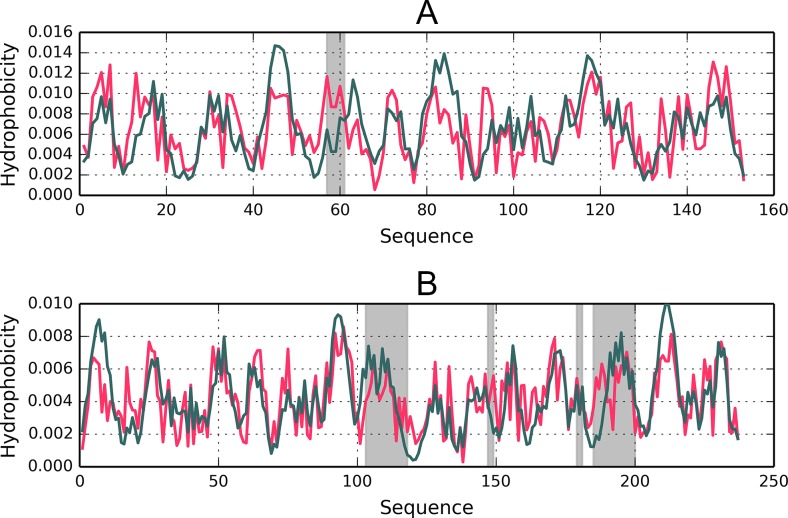
Hydrophobicity density distribution profiles (T – theoretical – green; O – observed – red) in 1A5Z-D1 and 1FW8-D2 (charts A and B respectively). Gray areas mark fragments where RD > 0.5

From the structural point of view both proteins provide examples of the so-called flavodoxin fold [35] with a centrally located β-propeller. Domain D1 (1A5Z) appears to include a well-ordered hydrophobic core while domain D2 (1FW8) lacks such a core, as indicated by its RD value, which is in excess of 0.5 (Table 2). It moreover turns out that the extra β-sheet (comprising three separate folds), which is not present in 1A5Z, diverges from the idealized distribution (Fig. 2). Eliminating this fragment from computations (an independent “droplike” capsule constructed for the remainder of the domain) produces a structure which is a good match for the theoretical model. Both domains exhibit similar properties in the scope of their central β-sheet, suggesting that the sheet contributes to structural stabilization (this is based on the assumption that the presence of a prominent hydrophobic core promotes tertiary stabilization). Both domains also contain a single helix (located at a similar distance from the propeller) which diverges from the model (Fig. 3 and Table 2).

Lactate dehydrogenase (1A5Z) is derived from the hyperthermophilic bacterium *Thermotoga maritima* and folds near the boiling point of water. Thermostable proteins are the focus of a separate study based on the fuzzy oil drop model (publication currently in preparation).

The catalytic residues in 1A5Z, as well as its disulfide bonds, are all located in domain 2 — thus, we will not consider them in the presented analysis.

Domain 2 of phospoglycerate kinase (1FW8-D2) exhibits RD > 0.5, which means that its hydrophobic core is deformed. The domain contains two β-sheets — a parallel sheet (labeled “I” in Table 2–133-140, 158–163, 205–207, 258–263, 294–298) and an antiparallel sheet (labeled “II” – 210, 223–229, 238–242). Such antiparallel conformation is not present in lactate dehydrogenase (1A5Z-D1). The β-structure which is analogous to 1A5Z is characterized by RD = 0.492, which implies local consistency with the theoretical hydrophobic core model.

As posited by the fuzzy oil drop model, ligand binding residues exhibit deviations from the theoretical status; this is because such residues interact with the ligand, which also participates in the formation of a shared core. In the case of strongly polar ligands, deviations may also be associated with the presence of additional structural elements not directly involved in forming the hydrophobic core, but instead distorting its structure.

In both presented proteins eliminating residues which participate in ligand binding results in a lower RD value. This means that the remainder of the molecule fulfills a stabilizing role by presenting a well-ordered hydrophobic core.

The exposed β-hairpin in D2 of 1FW8 may mediate interaction with other molecules present in the protein’s environment, or it may constitute a dynamic and unstable element (no prominent hydrophobic core) which also potentially affects interaction.

It furthermore appears that the α,β-superfold — particularly the twisted β-sheet — contains a hydrophobic core and therefore improves the stability of its parent domain.

### α,β-domain fold

The all-β-sheet superfold is represented by Cu, Zn superoxidase dismutase and concanavalin A. This type of all-β domain has the shape of a β-barrel. Both proteins appear to contain hydrophobic cores (RD < 0.5 in each case).

In 1B4L enzymatic and heavy metal ion binding residues are all located in loops. Table 3 lists positions adjacent to each fragment. The helical fold at 57–61 is locally discordant and contains a catalytic residue which binds Cu^2+^. Similarly, in 1CON the cadmium ion (Cd^2+^) is bound by a locally discordant β-fragment.

**Table 3:** RD values obtained for 1B4L and 1CON, for their respective secondary folds, for the entire β-sheet and for the fragment bounded by CYS residues which form a disulfide bond

Cu, Zn superoxide dismutase (1B4L) 2.60.40.200 – mainly β-sandwich			Concanavalin A (1CON) 2.60.120.200 – mainly β-sandwich		
Characteristics	Fragment	RD	Characteristics	Fragment	RD
Protein	153aa	0.433	Protein	237aa	0.416
	1–7 BI	0.065	I	3–11 B	0.407
	14–21 BI	0.403	I	14–18 H	0.421
	28–35 BI	0.468		23–30 B	0.355
I	41–48 BII	0.246		36–40 B	0.295
**E 63H, I**	**57–61 H**	**0.724**		46–56 B	0.333
I	82–89 BII	0.433		59–66 B	0.338
	95–101 BI	0.343		73–79 B	0.335
I	115–120 BII	0.257		80–84 H	0.218
	131–135 H	0.349	I	87–97 B	0.330
E 143R	145–148 BII	0.317		**103–118 B**	**0.601**
				122–130 B	0.135
				139–145 B	0.438
				**147–149 B**	**0.851**
				150–152 H	0.365
				153–156 B	0.384
				169–176 B	0.336
				**179–181 B**	**0.643**
			**I**	**185–200 B**	**0.762**
				208–217 B	0.337
				226–230 H	0.324
β-sheet		0.409	β-sheet		0.468
β -sheet I		0.368	Helices		0.446
β -sheet II		0.432			
SS-bond	57–146	0.431			

Values listed in boldface satisfy RD > 0.5. “B” and “H” stand for β-structural and helical forms respectively; “I” indicates an ion-binding fragment while “E” denotes that the given fragment contains an enzymatically active residue (listing its number and type).

Cu, Zn superoxide dismutase adheres to the theoretical hydrophobicity distribution model as a whole, although some secondary folds diverge from the model. The helical fragment at 57–61 is an example (RD > 0.5). Similarly, in 1CON, despite the overall adherence of the molecule and its β-sheet, four individual β-folds are found to be discordant.

**Fig. 4:**
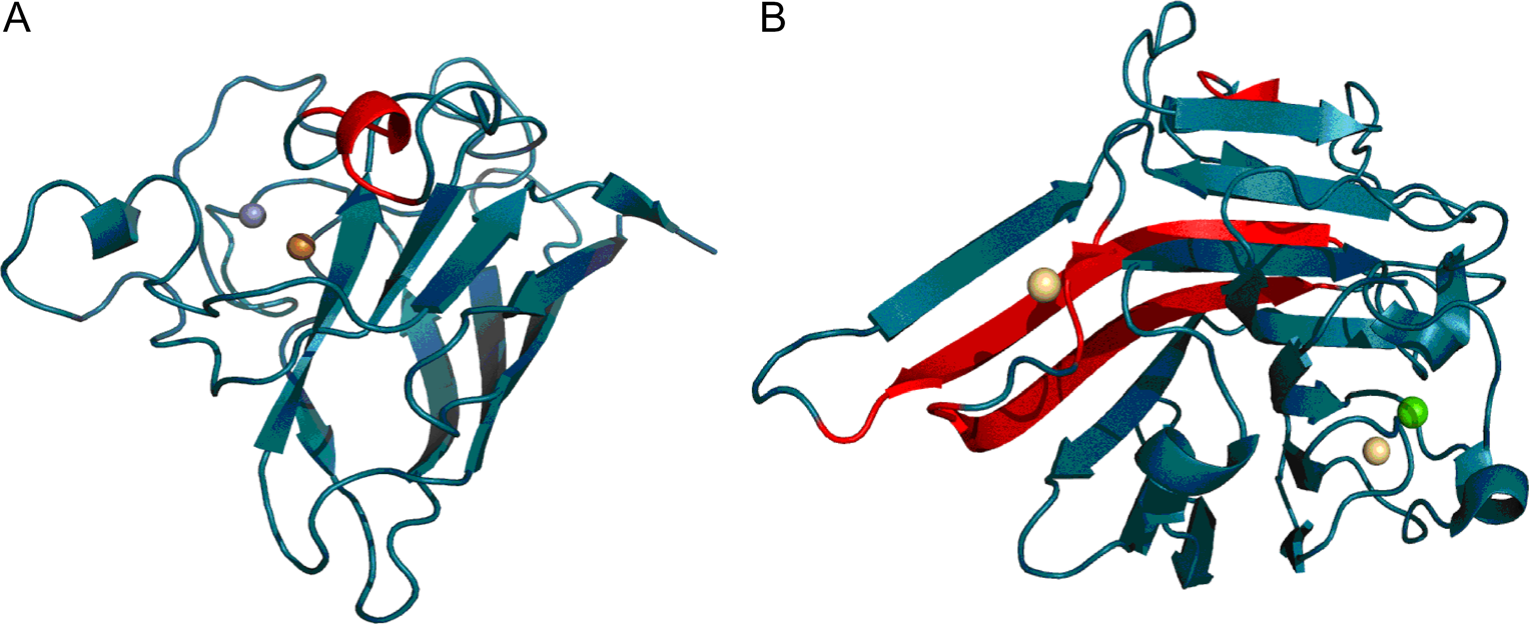
3D structures of 1B4L and 1CON, with fragments exhibiting high discordance (RD > 0.5) marked in red (Table 3). Spheres correspond to ion binding residues

**Fig. 5:**
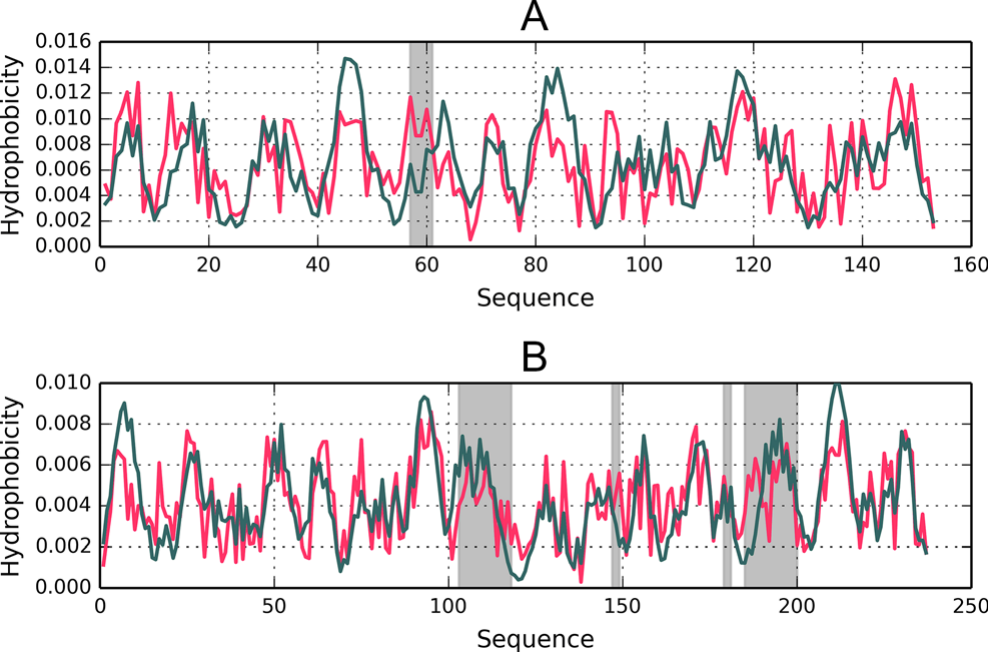
Hydrophobicity distribution charts for 1B4L (A) and 1CON (B). Gray bands mark divergent fragments

Both proteins (Fig. 4 and Fig. 5) exhibit highly ordered hydrophobic cores in spite of their biological diversity. Note that under the fuzzy oil drop model the concept of a “hydrophobic core” refers to a concentration of hydrophobicity density at the center of the molecule along with the presence of an encapsulating hydrophilic shell.

### All β-domain superfold

This category is represented by triose phosphate isomerase and pyruvate kinase domain 1. The core structure comprises a centrally located β-barrel where individual β-folds are interconnected by α-helices located outside of the fold. The sample proteins differ with respect to their composition and biological activity.

**Fig. 6:**
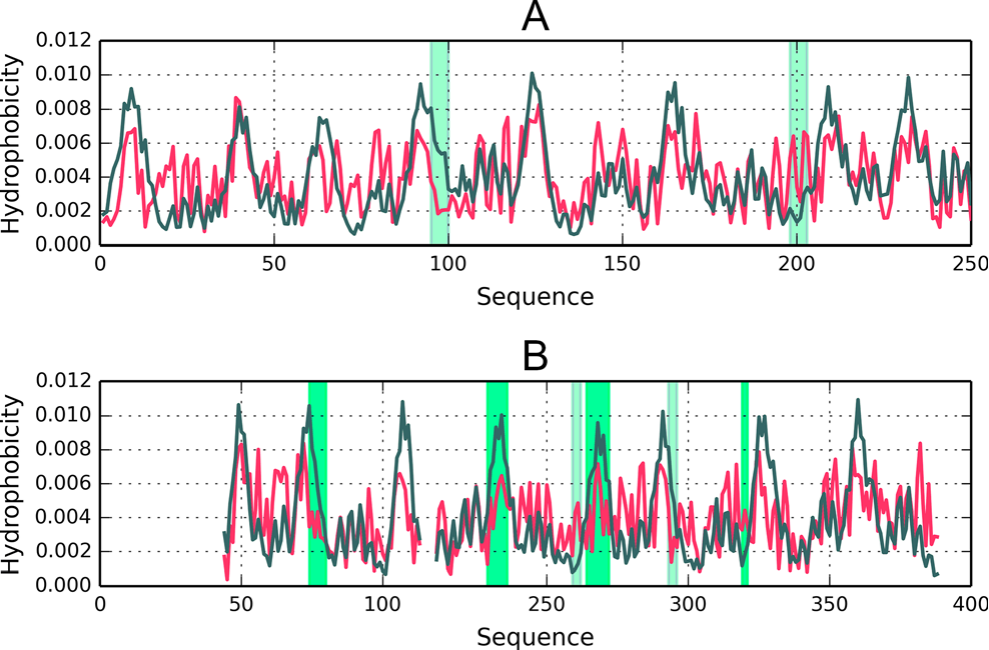
Hydrophobicity distribution profiles (theoretical – blue and observed – red) for 1AMK (A) with indication of eliminated fragments, along with 4DRS (B) with indication of eliminated fragments (blue) and the placement of ligand binding residues (green)

**Fig. 7:**
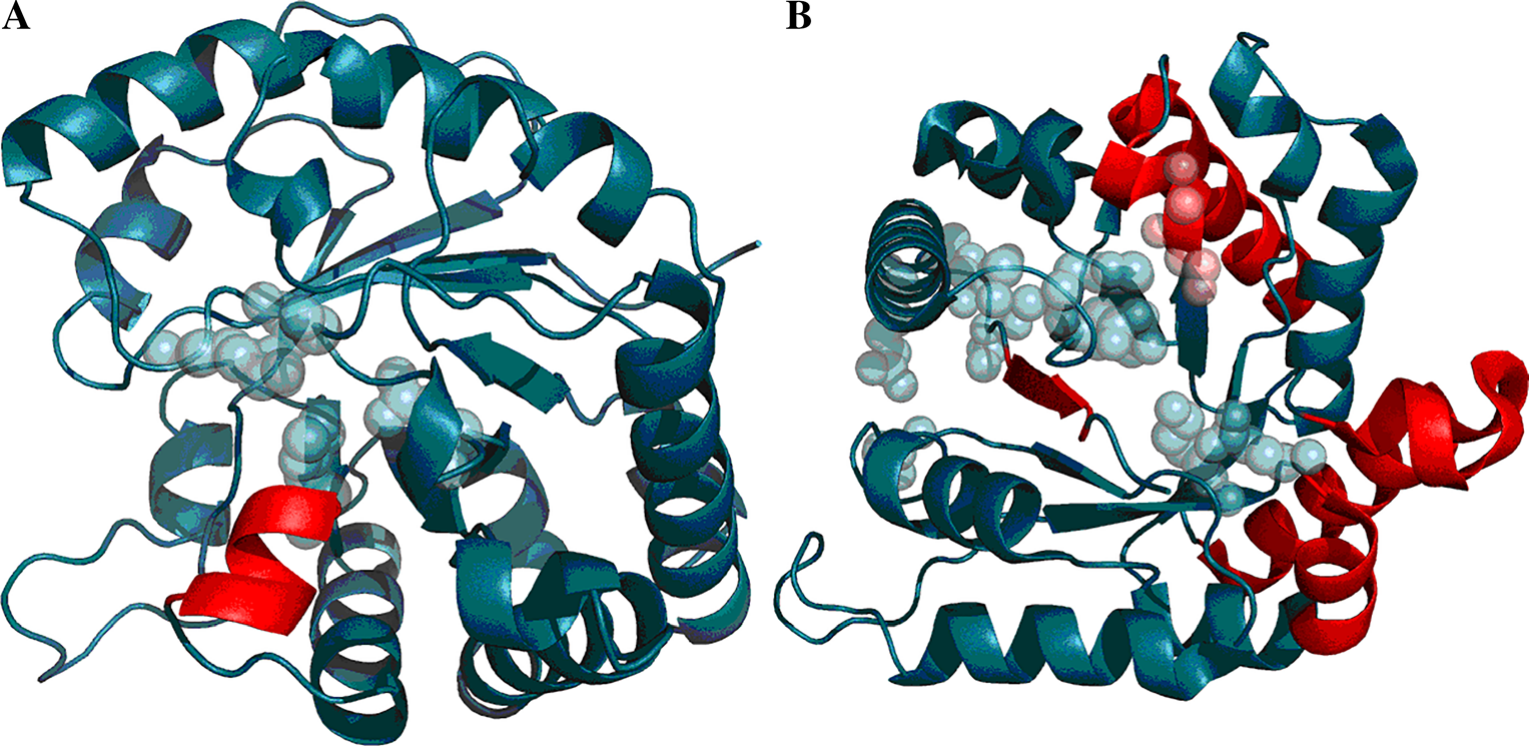
3D presentation of 1AMK (A – catalytic residues visualized with CPK; fragments exhibiting RD > 0.5 marked in red) and 4DRS (B – ligand-binding residues colored with CPK; eliminated residues colored pink; fragments exhibiting RD > 0.5 marked in red)

Elimination of catalytic residues (Table 4, Fig. 6, and Fig. 7) lowers the RD value to 0.479. This indicates local disorder in the area of the enzymatic active site.

**Table 4:** RD values obtained for 1AMK and 4DRS and for their respective secondary folds

Triose phosphate isomerase (1AMK) 1AMK – E.C.5.3.1.1			Pyruvate kinase – domain 2 (4DRS) E.C.2.7.1.40 – absent in domain 2		
Fragment	Characteristics	RD	Fragment	Characteristics	RD
**Protein**	**250**	**0.506**	**Protein**	**248**	**0.559**
6–12 B	E 11 N, 13 K,	0.401	47–52 B		0.330
17–30 H		0.402	53–57 H		0.278
38–42 B		0.452	58–69 H		0.475
**44–47 H**		**0.747**	71–77 B		0.497
48–55 H		0.336	81–98 H		0.432
59–63 B		0.263	**105–110 B**		**0.580**
79–86 H		0.330	214–224 H		0.404
89–93 B		0.148	231–235 B		0.378
**95–102 H**	**E 95H**	**0.634**	240–253 H		0.387
105–119 H		0.366	266–271 B		0.477
122–127 B		0.321	**273–279 H**		**0.551**
**130–136 H**		**0.541**	280–287 H		0.329
138–152 H		0.364	289–294 B		0.390
153–154 H		0.319	**296–301 H**		**0.647**
155–160 H		0.340	**302–305 H**		**0.534**
161–167 B	E 167E	0.317	**306–321 H**		**0.569**
**168–172 H**		**0.571**	323–327 B		0.395
179–198 H	E 173G	0.423	331–336 H		0.440
199–206 H		**0.654**	341–355 H		0.418
**207–212 B**		**0.542**	357–361 B		0.306
218–224 H		0.382	**362–367 H**		**0.603**
**229–233 B**		**0.524**	**370–387 H**		**0.553**
234–239 H		0.222			
**240–241 H**		**0.600**			
242–248 H		0.160			
Β-sheet			β-sheet		0.429
Helices			Helices		0.482
Eliminated	95–100,198–203	0.479	No ligand		0.546
No ligand		0.498	No residues	74–80 L, 229–236 L, 259–262, 264–272 L, 293–296, 319–321 L	0.486
NoE	11,13,95,167,173	0.500			

Values listed in boldface satisfy RD > 0.5. “B” and “H” stand for β- and helical forms respectively; “L” indicates a ligand-binding fragment while “E” denotes that the given fragment contains an enzymatically active residue (listing its number and type). The row labeled “NoE” represents the status of each domain following elimination of catalytic residues — the observed reduction in RD values indicates that catalytic residues diverge from the theoretical model.

Local deviations from the theoretical model caused by a ligand are commonplace. The ligand — by virtue of its presence — enforces a conformation which represents a balance between the protein’s own folding tendencies and the altered conditions introduced by the ligand’s polarized atoms. The end result of this process depends strongly on the ligand’s hydrophobicity [36].

Residues which diverge from the model, despite not taking part in ligand binding, may represent structurally encoded “readiness” for structural changes in the α/β domain.

Despite the observed differences, both domains share certain similarities. The helical fold at 95–100 in 1AMK exhibits a similar deviation from theoretical values when compared with the folds at 319–321 and 362–364 in 4DRS D2. It seems that local instability in all these areas may promote structural rearrangement.

### β-barrel

**Fig. 8:**
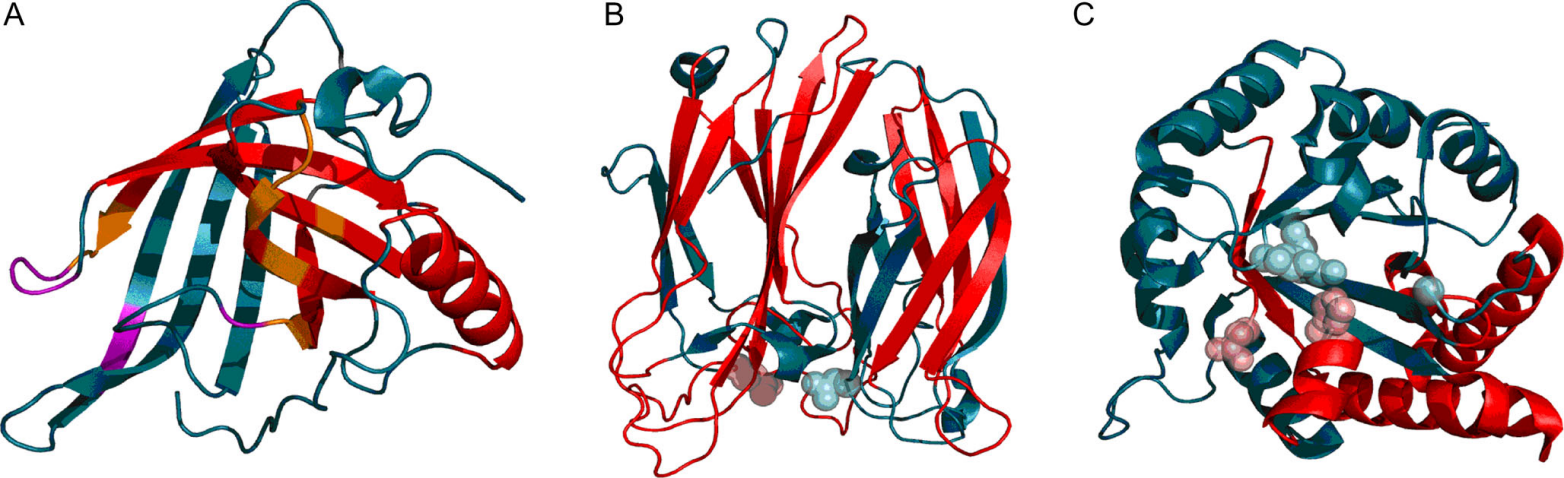
3D presentation of 1RBP (A), 1PNG (B), and 1TIM (C) with discordant fragments marked in red. Residues plotted with CPK represent catalytic residues

Three proteins representative of the β-barrel domain are characterized in Table 5 and Fig. 8.

**Table 5:** RD values representing individual secondary folds in 1RBP, 1PNG, and 1TIM

1RBP Up-and-down β barrel 2.40.128.20 – Mainly ββ-barrel Plasma retinol-binding protein precursor		1PNG Jelly roll EC 3.5.1.52 2.60.120.230 – Mainly ββ-sandwich Hydrolase		1TIM α/β barrel EC 5.3.1.1. 3.20.20.70 Αβ-Barrel isomerase	
Fragment	RD	Fragment	RD	Fragment	RD
Protein 175aa	**0.553**	Domain 2 135aa	**0.694**	Domain 247 aa	0.493
		**142–151 BI**	**0.771**	6–12 B E 11 N	0.435
		154–158 H	0.472	17–31 H	0.293
		159–161 BI	0.474	38–42 B	0.434
5–9 H	0.332	**162–168 L**	**0.605**	46–55 H	0.223
**22–30 B**	**0.567**	**169–175 BII**	**0.610**	**58–65 B**	**0.510**
39–48 B	0.431	176–180 L	0.427	79–87 H	0.418
52–63 B	0.406	**181–198 BI**	**0.688**	**90–93 B**	**0.590**
67–80 B	0.478	**199–201 L**	**0.560**	**95–103 HE 95H**	**0.617**
**84–93 B**	**0.589**	202–206 BI	0.187	105–119 H	0.361
**100–110 B**	**0.506**	207–209 L	0.415	123–129 B	0.141
**113–124 B**	**0.801**	210–217 BII	0.436	**130–137 H**	**0.725**
**128–139 B**	**0.787**	219–226 BII	**0.649**	**138–154 H**	**0.527**
**145–159 H**	**0.501**	227–229 L	**0.511**	159-167B E 165E	0.431
166–168 B	0.404	230–234 H	0.209	**168–170 H**	**0.670**
		**235–259 L**	**0.654**	**177–197 H**	**0.551**
		**260–264 BI**	**0.832**	**198–204 H**	**0.590**
		265–269 H	0.381	205–209 B	0.387
		**271–278 BII**	**0.742**	215–222 H	0.337
		**279–289 L**	**0.800**	227–231 B	0.476
		**290–303 BI**	**0.739**	232–237 H	0.221
		**304–310 L**	**0.578**	238–245 H	0.291
		**311–313 BII**	**0.508**		
**β-sheet**	**0.615**	**β - I**	**0.688**	β-sheet	0.438
**Helices**	**0.522**	**β - II**	**0.643**	**Helices**	**0.510**
**SS 4–160**	**0.566**	SS 51–56	0.195		
**SS 70–174**	**0.582**	**SS 203–208**	**0.680**		
		SS 231–252	0.483		
Removed: 29–31, 69–71 SS, 91–97 Loop, 117, 127–130 SS, 133–134 L	0.467			NoE	0.478

Values listed in boldface satisfy RD > 0.5. H – helix; B – β-fold; E # – placement of catalytic residue (with # corresponding to its code); L – ligand present as part of the described fragment

Each of the above listed proteins represents a different type of β-barrel. The assessment of hydrophobic core structure is, likewise, different in each case. It should be noted that the protein exists to fulfill a specific biological role and that its structure is a means to this end.

1RBP is a retinol binding protein. The presented domain contains three disulfide bonds which promote tertiary structural stability. Two of these bonds stabilize the domain in a “discordant” state, which is very likely their principal role.

In order to facilitate comparisons with other types of β-dominated supersecondary structures, we have included triole phosphate isomerase (1TIM) in our study. The corresponding form is an α/β barrel consisting of eight pairs of alternating β-strands and α-helices rolled up to form an inner barrel of eight parallel β-strands surrounded by an outer barrel of eight parallel helices. Analysis based on the fuzzy oil drop model indicates the presence of a well ordered hydrophobic core. Stabilization is provided by the arrangement of β-folds (RD = 0.493), while catalytic residues deform the domain (their elimination results in RD = 0.478).

Discordant helixes include the fragments at 95–103 (RD = 0.617), 130–137 (RD = 0.725), 138–154 (RD = 0.527), 168–170 (RD = 0.671), 177–197 (RD = 0.551), 198–204 (RD = 0.590). Catalytic residues are located in direct proximity to the active site. Experience with the fuzzy oil drop model indicates that enzymatic active sites are, in most cases, deficient in terms of hydrophobicity and diverge significantly from theoretical values. In the presented cases catalytic residues belong to the helices at 95–103, 168–170, 177–197, and 198–204, with RD values in excess of 0.5 in all cases.

### Miscellaneous

The arrangement of helical and β-folds in higher-order structures varies greatly both with respect to the quantity of each and their conformation. An example of a purely α protein is provided by the b562 cytochrome (1APT) from *E. coli*. Topologically, this protein is a sequence of antiparallel helical fragments described as an up-down-up-down 4-helix bundle.

In contrast, the Fab IgG immunoglobulin domain (7FAB) is comprised almost entirely of β-folds, with its structure characterized as a sandwich of 3- and 4-stranded antiparallel β-sheets.

A combination of helical and β-motifs is found in the 163-residual domain of dogfish lactate dehydrogenase (6LDH), which contains a 6-stranded parallel β-sheet with crossovers between β-strands and α-helices forming a right-handed helical turn with flanking β-strands.

The cytochrome is characterized by RD = 0.426, suggesting the presence of a well-ordered hydrophobic core. All its helical fragments also satisfy RD < 0.5 (3–20: RD = 0.355; 23–43: RD = 0.481; 45–49: RD = 0.342; 57–81: RD = 0.402; 82–94: RD = 0.408). It seems that this protein depends strongly on the stabilizing influence of hydrophobic interactions. Given that the protein’s biological role is to bind heme, its structure provides suitable conditions for firm anchoring of the ligand at its center, with the polypeptide chain forming an “envelope” around the heme molecule.

### 7FAB

The Fab IgG domain fragment consists of two chains: the light chain (L) and the heavy chain (H), each of which is further composed of two domains (labeled V and C). All these structures exhibit a similar conformation, often referred to as an immunoglobulin-like domain: a sandwich comprising two β-sheets which we will refer to as the upper core and lower core respectively.

Under the CATH classification the Fab fragment is characterized as 2.60.40.10 mainly β-sandwich. Both cores are linked by a centrally placed disulfide bond. The biological role of the Fab fragment is to recognize antigens via so-called CDRs — short variable loops which interact with the antigen, triggering a process called immunological signal transduction. This produces structural changes in the Fc fragment of immunoglobulin, priming it for complexation of the C1q complement, which is a first step in a cascade of reactions resulting in destruction of the cell which originally presented the antigen.

The role of the Fab fragment is therefore to bind the antigen and trigger a signaling pathway. Both processes require a flexible structure, capable of accommodating the antigen and producing structural changes elsewhere in the molecule. The flexibility must, however, remain selective so as to ensure that the correct ligand is recognized and the correct signal sent.

**Fig. 9:**
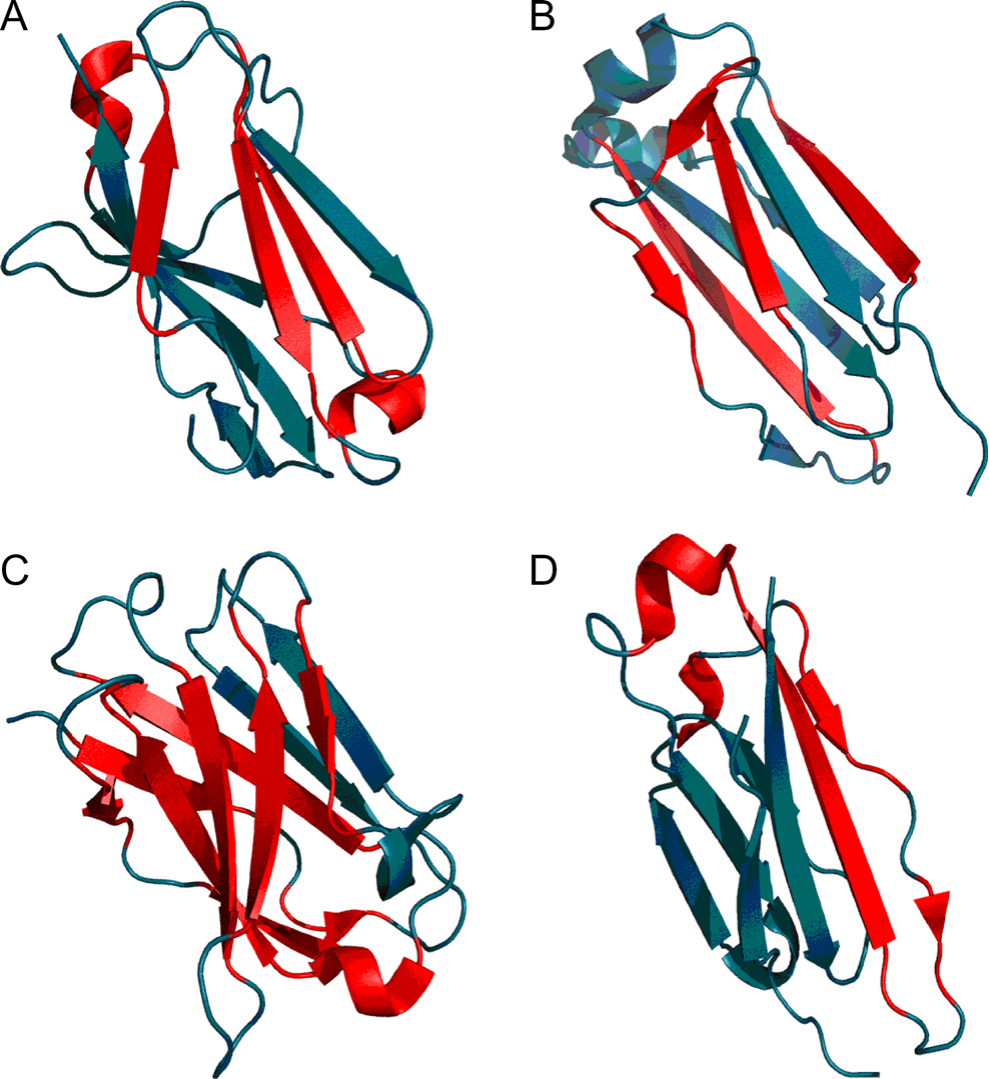
3D representation of 7FAB immunoglobulin domains: VL (A), CL (B), VH (C), and CH (D). Fragments marked in red satisfy RD > 0.5

This situation is evidenced by the RD status of individual β-folds in all four domains: VH, CH1, VL, and CL (see Table 6 and Fig. 9).

**Table 6:** RD values calculated for ragmentdomains of the Fab fragment of immunoglobulin G, revealing variable stability of individual secondary folds

Chain L				Chain H			
Domain VL		Domain CL		Domain VH		Domain CH1	
Fragment	RD	Fragment	RD	Fragment	RD	Fragment	RD
**Domain**	**0.584**	Domain	0.320	**Domain**	**0.601**	**DOMAIN**	**0.538**
**8–12 BI**	**0.584**	110–114 BI	0.073	**3–7 BI**	**0.583**	124–128 BI	0.293
**18–24 BII**	**0.649**	117–123 H	0.388	**10–12 BII**	**0.673**	140–149 BI	0.424
**28–32 H**	**0.629**	126–135 BI	0.254	**18–25 BI**	**0.682**	155–159 BII	0.294
36–40 BI	0.437	**141–147 BII**	**0.554**	**33–39 BII**	**0.528**	**160–162 H**	**0.670**
47–49 BI	0.281	**149–151 BII**	**0.545**	**46–52 BII**	**0.551**	**166–170 BI**	**0.808**
56–62 BII	0.369	**154–158 BI**	**0.525**	**56–60 BII**	**0.702**	**173–175 BI**	**0.766**
**64–71 BII**	**0.703**	161–163 BI	0.083	62–64 H	0.002	**179–188 BI**	**0.878**
**74–78 H**	**0.675**	**167–176 BI**	**0.555**	66–72 BI	0.330	**189–193 H**	**0.556**
79–87 BI	0.468	177–183 H	0.353	76–83 BI	0.358	198–204 BII	0.283
90–94 BI	0.358	186–193 BII	0.394	**86–90 H**	**0.874**	205–208 H	0.492
97–102 BI	0.710	**196–202 BII**	**0.543**	**91–99 BII**	**0.665**	209–215 BII	0.367
				**103–108 BII**	**0.576**		
				**111–116 BII**	**0.689**		
**SS 22–86**	**0.600**	SS 130–189	0.332	**SS 22–95**	**0.568**	**SS 144–200**	**0.638**
**B - I**	**0.613**	B - I	0.331	B - I	0.481	**B - I**	**0.648**
B - II	0.477	B - II	0.482	**B - II**	**0.703**	B - II	0.242

Values listed in boldface satisfy RD > 0.5.

The variable status of individual secondary folds comprising Fab domains suggests local instabilities required for complexation of antigens. One of the domains (CL) is stable as a whole and includes a stable β-I sheet, while all other domains are characterized as relatively unstable and therefore flexible. The stabilizing influence of SS bonds likewise varies: only in the CL domain does the affected fragment remain consistent with theoretical predictions.

Immunoglobulins work in an unpredictable environment — the antigen is never known a priori. Thus, a system characterized by variable local stability creates suitable conditions for targeted signaling while preserving the immunoglobulins ability to interact with a variety of antigens.

A review of immunoglobulin-like domains (which includes domains not associated with immunoglobulin activity, including enzymes and structural proteins) can be found in [8]. The authors reveal a specific arrangement of accordant and discordant fragments comprising the domain, which predispose it toward specific structural changes. Overall, the domain is characterized by poor stability, evidenced by locally high RD values.

**Fig. 10:**
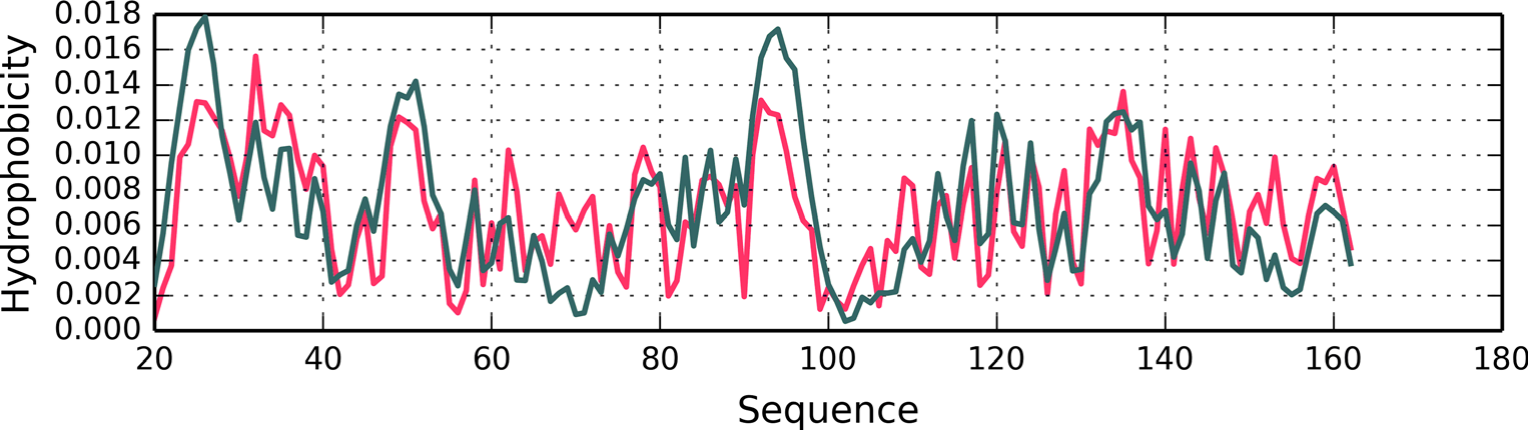
N-terminal domain of dogfish lactate dehydrogenase (6LDH) – theoretical (T – blue) and observed (O – red) distributions indicating the presence of a prominent hydrophobic core

**Fig. 11:**
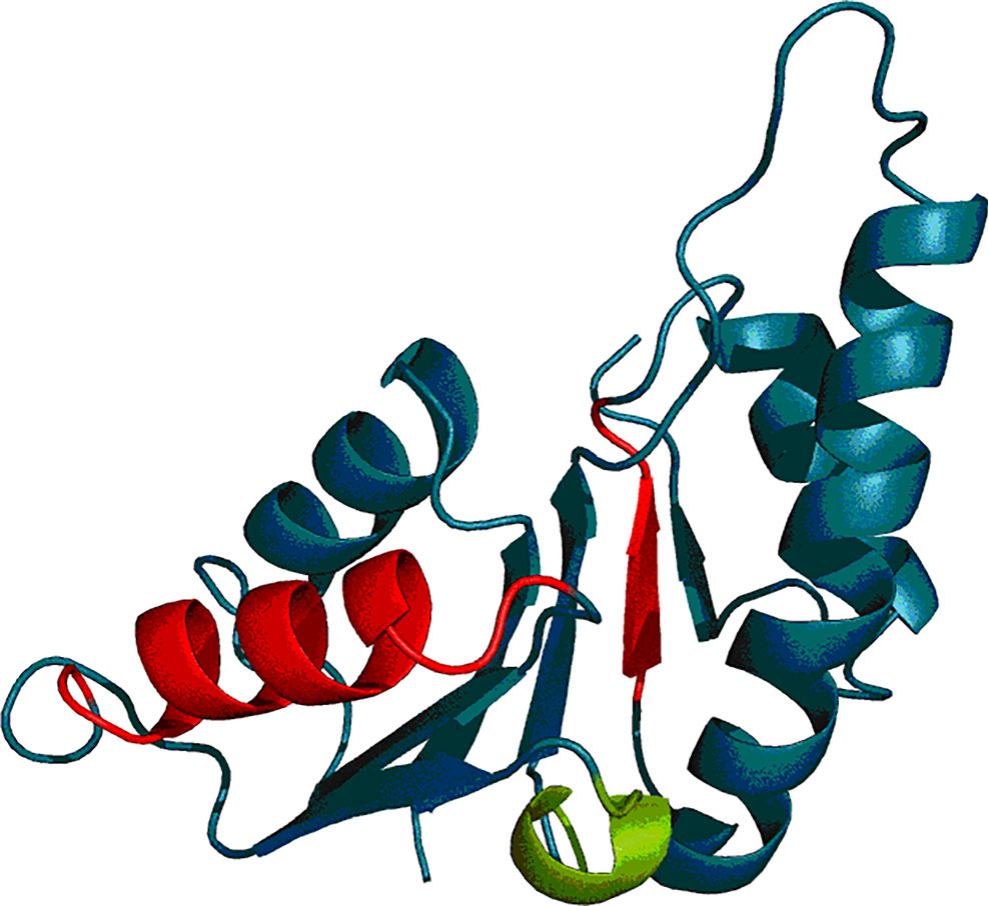
3D representation of the N-terminal domain (20–162) of 6LDH. Fragments marked in red exhibit RD > 0.5 while the fragment marked in green corresponds to the right-handed helical fold causing β-strand flanking

The dogfish lactate dehydrogenase N-terminal 163-aa fragment is an example of a 6-strand parallel β-structure linked by a right-handed helical turn. The domain as a whole is characterized by RD = 0.489, while elimination of its N-terminal fragment (1–19 aa), which significantly disrupts the globular form, further reduces RD to 0.428. Evidently, the domain contains a prominent hydrophobic core. Table 7 lists the RD status of its individual folds (compare also Fig. 10 and Fig. 11).

**Table 7:** RD values calculated for the N-terminal domain of dogfish lactate dehydrogenase (6LDH)

6LDH	
Fragment	RD
Domain 20–162	0.428
21–27 B	0.146
29–41 H	0.241
47–52 B	0.293
**54–68 H**	**0.525**
76–80 B	0.337
82–87 H	0.447*
90–95 B	0.214
105–128 H	0.447
**132–136 B**	**0.559**
139–152 H	0.349
157–161 B	0.332
β-sheet	0.366
HELICES	0.396

Values listed in boldface satisfy RD > 0.5. The asterisk marks the right-handed helical fold which causes β-strand flanking

### Cytochrome fold

The author of [1] also addresses the concept of protein families, with cytochromes discussed as a representative example. This family comprises both eukaryotic cytochromes and c-type cytochromes present in prokaryotes. Both fulfill a similar biological role, acting as electron carriers. Cytochromes derived from various organisms are sequentially dissimilar despite retaining similar 3D forms. The conclusion presented in [1] is that evolution preserves the structural and functional properties of proteins rather than their sequential arrangement. We further propose that the subject of evolutionary conservation is the structure of the proteins hydrophobic core — as we attempt to substantiate in this part.

Table 8 and Table 9 present the properties of cytochromes under consideration. The FOD status of the entire molecule appears preserved despite differences in chain length (88–135 aa) along with the placement and conformation of individual secondary folds. “No ligand” indicates parts of the molecule which do not contact the ligand. Evidently, such fragments are also characterized by consistent RD values.

**Table 8:** RD values calculated for cytochromes c_550_ and c_555_

4 J20 Cytochrome c_555_ *Chlorobium tepidum* 1.10.760.10 mainly α orthogonal bundle		155C Cytochrome c_550_ *Paracoccus* 1.10.760.10 mainly α orthogonal bundle	
Fragment	RD	Fragment	RD
Protein	0.356	Protein	0.399
4–16 H*	0.434	5–13 H	0.278
17–21 H*	0.336	14–17 L*	0.368
23–27 H*	0.409	18–20 B*	**0.835**
28–31 L*	0.585	21–34 L*	0.370
32–41 H*	0.463	**35–38 B**	**0.661**
43–54 H*	0.377	39–54 L*	0.306
55–57 B*	0.338	55–65 H*	0.198
60–62 B*	0.452	66–71 L*	0.385
63–68 H*	**0.595**	72–81 H*	0.385
72–86 H*	0.119	82–105*	0.439
		106–118 H	0.277
β-strand 55–62	0.290	**β-strand 18–38**	**0.554**
β-strand (55–57) + (60–62)	0.298	**β-strand (18–20) + (35–38)**	**0.706**
Ligand	0.338	Ligand	0.549
No ligand	0.356	No ligand	0.457

Values listed in boldface satisfy RD > 0.5. Asterisks mark fragments involved in interaction with the ligand. The row labeled “Ligand” corresponds to ligand-binding residues, while the row labeled “Noligand” presents residues not involved in ligand interaction.

**Table 9:** RD values calculated for cytochromes b_562_, c_2_ and c

256B *Ecoli* 1.20.120.10 mainly α up-down bundle Cytochrome b_562_		1JDL *Rhodospirillum rubrum* 1.10.760.10 α orthogonal bundle Cytochrome c_2_		2C2C *Rhodopsin rubrum* 1.10.760.10 mainly α orthogonal bundle Cytochrome c_2_		5CYT *Thunnus alalunga* 1.10.760.10 mainly α orthogonal bundle Cytochrome c	
Fragment	RD	Fragment	RD	Fragment	RD	Fragment	RD
Protein	0.411	Protein	0.476	Protein	0.448	Protein	0.392
2–20 H	0.341	4–12 H	0.295	**3–11 H**	**0.503**	2–14 H*	0.312
22–41 H*	**0.543**	13–15 H*	0.229	12–15 H*	0.359	15–48 L*	0.368
45–49 H*	0.371	16–49 L*	0.402	16–48 L*	0.488	49–54 H*	0.442
55–81 H*	0.424	50–59 H*	**0.506**	49–58 H*	0.202	55–59 L*	**0.643**
83–92 H	0.300	60–63 L*	**0.777**	59–62 L*	**0.710**	60–70 H*	0.254
93–106*	0.377	64–72 H*	0.211	63–73 H*	0.345	71–75 H*	0.296
		**74–86 H**	**0.661**	74–83 H	0.493	76–86 L*	0.442
		88–93 H	0.446	84–96 L*	**0.561**	87–102 H*	0.233
		94–103 L*	0.487	97–109 H	0.192		
		104–116 H	0.236				
		117–119	0.146				
Ligand	0.225	Ligand	0.549	Ligand	0.593	Ligand	0.490
No ligand	0.408	No ligand	0.457	No ligand	0.424	No ligand	0.376

Values listed in boldface satisfy RD > 0.5. Asterisks mark fragments involved in interaction with the ligand. The row labeled “Ligand” corresponds to ligand-binding residues, while the row labeled “No ligand” presents residues not involved in ligand interaction.

**Fig. 12:**
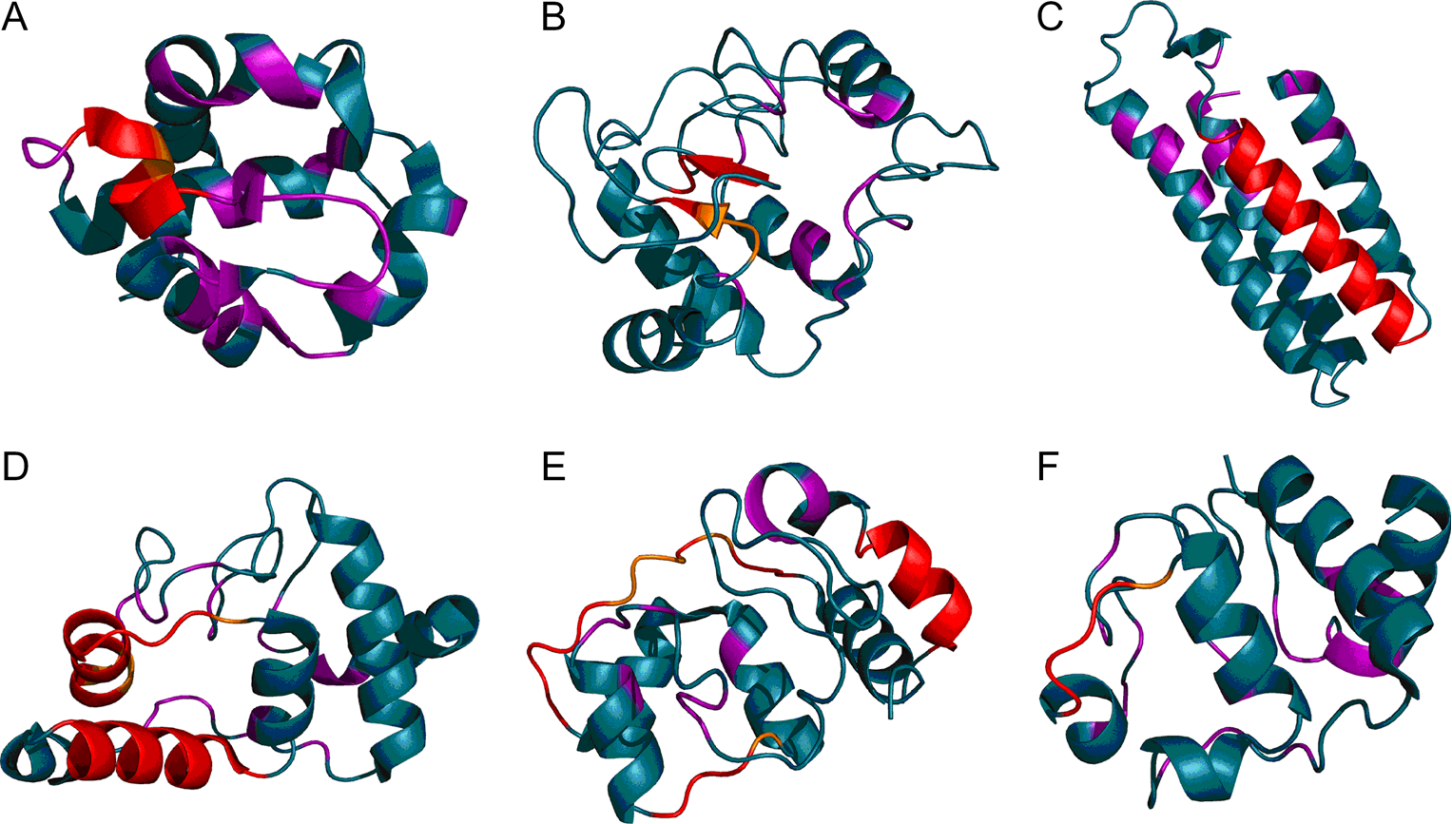
3D representation of cytochromes with fragments exhibiting RD > 0.5 marked in red: 4 J20 (A), 155C (B), 256B (C), 1JD2 (D), 2C2C (E), and 5CYT (F). Pink fragments are involved in interaction with the ligand (heme)

Cytochromes are proteins which act as “envelopes” for their ligand (heme – see Fig. 12). This ligand is a large, flat molecule dominated by hydrophobic interactions. Comparative analysis of cytochromes boils down to comparing various forms of “envelopes” which create suitable conditions for maintaining the intended level of oxidation of iron ions and expose them for participation in biological processes. These forms are briefly outlined in Tables 8 and 9. The status of the fragment which does not participate in binding heme appears not to change. This is due to its primary role — structural stabilization. In contrast, heme-binding residues exhibit variable FOD status, which may be related to the process of exposing the ligand for various types of interactions.

The conclusions of [1] also highlight the high structural similarity of the NAD^+^ binding fragment in enzymes which participate in many different metabolic pathways. These proteins are more widely discussed in [8].

## Discussion and conclusions

Applying the fuzzy oil drop model to structures classified using the supersecondary fold criterion reveals similar FOD characteristics, with hydrophobic cores observed either on the domain or on the protein level. A global analysis of a broad nonredundant set of proteins derived from the PDB database indicates that most domains generate prominent hydrophobic cores (RD < 0.5) [37]. It seems that, at least on the level of domains, the water environment greatly influences the folding process.

It is up to each researcher to select fragments of interest, depending on the problem being studied. In our experience, fragments which diverge from theoretical predictions produced by the FOD model are typically associated with biological function. The status of particular secondary fragments in supersecondary structures may quantitatively reveal their contribution to overall supersecondary stabilization.

In summary, we can state that the targeted stability required in many biological processes can be supplied by a combination of nonbinding interactions, structural stabilization mediated by hydrophobic forces, and the presence of disulfide bonds. As shown in [38], SS-bonds may either reinforce or disrupt hydrophobic core stability. The influence of the water environment on protein folding also explains the mechanism behind hydrophobic collapse, which reduces the protein’s conformational space sufficiently to enable a random search for the native fold [39]; this shows that hydrophobic collapse is sufficient to solve Levinthal’s paradox [40].

Discussions concerning the fundamental role of the hydrophobic core have a long history [41] with both experimental [42] and theoretical approaches [43].

In the context of these observations and the analysis presented in this paper we can conclude that the answer to the question posed in the title is “yes”. We can furthermore state that the fuzzy oil drop model is able to describe the hydrophobic core structure in both qualitative and quantitative terms including the substantial and specific difference of hydrophobicity distribution observed in amyloids [31].
